# Downregulation of GRP78 and XIAP is correlated with apoptosis during cerulein-induced acute pancreatitis in rats via regulation of caspase activation

**DOI:** 10.3892/mmr.2012.1241

**Published:** 2012-12-18

**Authors:** YONG LIU, ZONG-GUANG ZHOU, BING ZHOU, RONG WANG, HUI YAN, YUAN LI

**Affiliations:** 1 Department of Gastroenterological Surgery, West China Hospital, Sichuan University, Sichuan, P.R. China; 2 Institute of Digestive Surgery and State Key Laboratory of Biotherapy, West China Hospital, Sichuan University, Sichuan, P.R. China

**Keywords:** acute pancreatitis, 78-kDa glucose-regulated protein, X-linked inhibitor of apoptosis protein, apoptosis, caspase

## Abstract

Our aim in the present study was to investigate the potential roles of the 78-kDa glucose-regulated protein (GRP78) and the X-linked inhibitor of apoptosis protein (XIAP) in the regulation of apoptosis during cerulein-induced acute pancreatitis (CAP). A rat CAP model was induced by injection of cerulein (50 μg/kg), and the severity of CAP was estimated by measuring serum amylase and lipase, pancreatic edema and histological changes. Pancreatic acinar cell apoptosis was determined by terminal-deoxynucleotidyl-transferase-mediated dUTP nick-end labeling (TUNEL) assay, and the expression of GRP78, XIAP and the apoptotic genes caspase-3, -7 and -9 were determined by real-time quantitative PCR and western blotting. After induction with cerulein, increased serum amylase and lipase, pancreatic edema, inflammation and apoptosis were observed in CAP rats. Furthermore, the mRNA and protein levels of GRP78 and XIAP were significantly downregulated in CAP rats, while the mRNA levels of caspase-3, -7 and -9, as well as the cell apoptotic index were markedly increased when compared with control rats (P<0.05). The expression of GRP78 and XIAP was negatively correlated with caspase expression in CAP (P<0.05). This study suggests that the downregulation of GRP78 and XIAP were correlated with apoptosis in pancreatic acinar cells, and that this may occur through the regulation of caspase activation during CAP.

## Introduction

Acute pancreatitis (AP) is an inflammatory disorder of the exocrine pancreas, which carries considerable morbidity and mortality, and the pathophysiology of which remains unknown (1,2). Parenchymal cell death is a major complication of AP (3). Several recent studies have indicated that pancreatic acinar cell death occurs through both necrosis and apoptosis, and the severity of AP directly correlates with the extent of necrosis and inversely with that of apoptosis (3–5). Apoptosis is the process of programmed cell death, during which no inflammatory reaction occurs. By contrast, necrosis is a form of premature cell death, which is capable of leading to the release of a variety of inflammatory agents. The reported elevation of pancreatic acinar cell apoptosis may indicate a beneficial response following the onset of pancreatitis (6–8).

There are two main apoptotic pathways. The extrinsic (death receptors) and the intrinsic (mitochondrial) pathways are activated by caspase-8 and caspase-9, respectively. Activated caspase-8 and -9 subsequently cleave and activate the executioner caspases, such as caspase-3 and caspase-7, which then cleave intracellular substrates, resulting in apoptosis (9–11). By contrast, caspase activation may be tightly controlled by endogenous inhibitors, such as the inhibitor of apoptosis proteins (IAP), a family of structurally related proteins that negatively regulate caspase activation. The X-linked IAP (XIAP) is the most potent of the eight mammalian IAPs and blocks cell apoptosis through inhibiting the activation of caspases-3, -7 and -9 (12,13).

The 78-kDa glucose-regulated protein (GRP78), also known as BiP or HSPA5, is not only a major molecular chaperone but also an anti-apoptotic molecule of the cell death signaling pathway (14–16). Previous studies have indicated that GRP78 plays an anti-apoptotic role, possibly through interaction with XIAP (17,18). In addition, GRP78 exhibits anti-apoptotic properties through interference with caspase activation and release (14,19). However, the pathological role of GRP78 and XIAP in AP remains unclear.

It is unknown whether GRP78 and XIAP participate in the pathogenesis of AP and whether GRP78 and XIAP correlate with the regulation of cell death during AP. The aim of the present study was to investigate the expression of GRP78 and XIAP and their potential roles in the regulation of apoptosis during CAP in rats.

## Materials and methods

### Animal experiments

Male Wistar rats (Experimental Animal Center of Sichuan University, Chengdu, China), weighing 200 to 220 g, were used in this study. All the animals were maintained at 23°C on a 12-h light/dark cycle and starved for 12 h prior to experimentation, but were allowed free access to water. All the animal experiments were conducted according to the guidelines of the local animal use and care committees and executed according to the National Animal Welfare Law of China. The rats were randomly divided into 2 groups: the cerulein-treated AP group (CAP) and the saline-treated control group. The CAP group was further divided into 5 subgroups depending on the time of sampling: 1, 3, 6, 12 or 24 h (n=6). Acute pancreatitis was induced in the CAP group of rats by injecting cerulein (Sigma, St. Louis, MO, USA) at a dose of 50 μg/kg intraperitoneally four times at 1-h intervals. Normal saline was substituted for cerulein with the same volume for the controls. After the last injection, rats were sacrificed at 1, 3, 6, 12 and 24 h under 1% pentobarbital anesthesia (50 mg/kg of body weight). Fresh pancreatic tissue was removed, weighed immediately for the pancreatic tissue edema assay, rinsed in TRIzol (Gibco, Carlsbad, CA, USA) for subsequent RNA isolation, frozen at −80°C for western blot analysis, and fixed with 10% formaldehyde prior to paraffin sectioning for histological examination or terminal-deoxynucleotidyl-transferase-mediated dUTP nick-end labeling (TUNEL) assay. Inferior vena cava blood was collected, centrifuged at 4°C, and the serum was stored at −80°C for enzyme linked immunosorbent assay (ELISA).

### Serum amylase and lipase determination

Serum amylase and lipase activity were determined by commercially available ELISA kits (R&D, Minneapolis, MN, USA), according to the manufacturer’s instructions.

### Pancreatic tissue edema assay

For the evaluation of pancreatic tissue edema, the pancreas was removed and immediately weighed. It was then dried in an oven at 80°C for 72 h, and reweighed. The extent of pancreatic edema was determined by measuring tissue water content (wet weight - dry weight/wet weight × 100 = percentage tissue water content).

### Histological examination

For routine histological examination, 4-μm sections of 10% formalin-fixed, paraffin-embedded tissue were prepared and stained with hematoxylin and eosin (HE). All microscopic sections were evaluated in a blind fashion.

### TUNEL assay

In the pancreatic tissue, apoptosis was detected by use of the *in situ* cell death detection kit (Roche Applied Science, Mannheim, Germany), according to the manufacturer’s instructions. Briefly, tissue was fixed in 10% buffered formaldehyde, embedded in paraffin, and 4-μm sections were adhered to glass slides. After dewaxing and rehydration, the sections were incubated with TUNEL reaction mixture at 37°C for 1 h. Finally, the sections were analyzed under a fluorescence microscope (Olympus, Tokyo, Japan) at 450–500 nm. Images of these tissues were obtained using an image acquisition system (Olympus DD70 BX51). TUNEL-positive cells displayed brilliant green fluorescence. For each test, negative controls were included. Apoptotic index (AI) was determined as the percentage of TUNEL positive cells in 10 randomly selected high-power fields by averaging 10 counts per tissue section.

### Quantitative real-time reverse transcriptase PCR

Fresh pancreatic tissue (50–80 mg) was collected per rat, and total RNA was isolated using TRIzol (Gibco). Total RNA (5 μg) was reverse transcribed and 1 μg of the RT product was subjected to PCR in the presence of specific primers. The sequences of the primers were as follows: (*GRP78*: 5′-GAA ACTGCCGAGGCGTAT-3′/5′-ATGTTCTTCTCTCCCTCT CTCTTA-3′; *Caspase-3*: 5′-CGGACCTGTGGACCTGAA A-3′/5′-GGGTGCGGTAGAGTAAGC-3′; *Caspase-7*: 5′-TCT ATGTGCCCCGTCAGTA-3′/5′-ACATCCATACCTGTCGC TTT-3′; *Caspase-9*: 5′-ACGACCTGACTGCTAAGAAA-3′/5′-AGCCATGAGAGAGGATGAC-3′; *XIAP*: 5′-TGTGAG TGCTCAGAAAGATAAT-3′/5′-TGCTTCTGCACACTG TTTACA-3′; *β-actin*: 5′-CGTGAAAAGATGACCCAGAT-3′/5′-ACCCTCATAGATGGGCACA-3′). Conditions for all PCRs were optimized on an iCycler IQ (Bio-Rad, Hercules, CA, USA) system for a 30-μl reaction using the following 40 cycle program: 94°C for 20 sec, 53°C for 30 sec, and 72°C for 30 sec. All samples were amplified simultaneously in triplicate in one assay-run. β-actin was included in each reaction as an internal standard and relative quantitative gene expression was calculated using the 2^−ΔΔCt^ method as described previously (20).

### Western blot assay

Total proteins were prepared from pancreatic tissue using a total protein extraction kit (KeyGen Biotech., Co., Ltd., Nanjing, China) and the concentrations were determined using a bicinchoninic acid protein assay kit (Pierce, Rockford, IL, USA). Each 20-μg aliquot of total protein was loaded onto a 12% sodium dodecyl sulfate-polyacrylamide gel for electrophoresis, and then transferred onto polyvinylidene difluoride membranes (Millipore, Billerica, MA, USA). Following complete protein transfer, the membranes were blocked with 5% milk powder solution for 2 h and incubated with primary antibodies overnight. The primary antibodies used in this study included polyclonal GRP78 (Abcam, Cambridge, UK) and monoclonal XIAP (Cell Signaling, Beverly, MA, USA) in a 1:1,000 dilution individually. For internal reference, a monoclonal rabbit anti-rat β-actin antibody (1:1,000 dilution; Cell Signaling) was used. After washing the membranes, the goat polyclonal anti-rabbit immunoglobulin G secondary antibody (Cell Signaling) conjugated to horseradish peroxidase was applied in a 1:5,000 dilution and incubated for 2 h at room temperature. Finally, antibody binding was visualized using the enhanced chemiluminescence system (Pierce), and the semi-quantitative grayscale intensity was generated with Multi Gauge V3.0 software (Fujifilm, Tokyo, Japan).

### Statistical analysis

The experimental data are expressed as the means ± SE. The differences between 2 groups were compared by the non-paired Student t-test. Pearson correlation coefficient was calculated where indicated. P<0.05 was considered to indicate a statistically significant difference. All tests were performed using the statistical package SPSS software 13.0 (SPSS, Chicago, IL, USA).

## Results

### Evaluation of CAP

**Figure 1:**
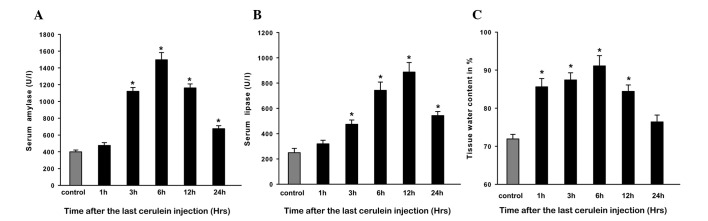
Cerulein treatment induced biochemical changes in the pancreas. Serum (A) amylase and (B) lipase were detected by ELISA. (C) Pancreatic edema was determined by tissue water content assay. Values are the means ± SE (n=6). ^*^P<0.05 vs. control. ELISA, enzyme-linked immunosorbent assay.

In this study, intraperitoneal injections of 50 μg/kg cerulein led to the induction of AP, and the severity of CAP was assessed by serum amylase and lipase levels and pancreatic edema. Compared with control rats, amylase and lipase levels in CAP rats were significantly increased within 3 h and peaked at 6 and 12 h, respectively (P<0.05), then a trend of return to baseline levels was observed at 24 h (Fig. 1A and B). Injections of cerulein induced prominent pancreatic edema as assessed by tissue water content, which showed that tissue water content was significantly increased at 1 h and peaked at 6 h; thereafter it gradually decreased and returned to baseline at 24 h (Fig. 1C).

### Histological findings

**Figure 2:**
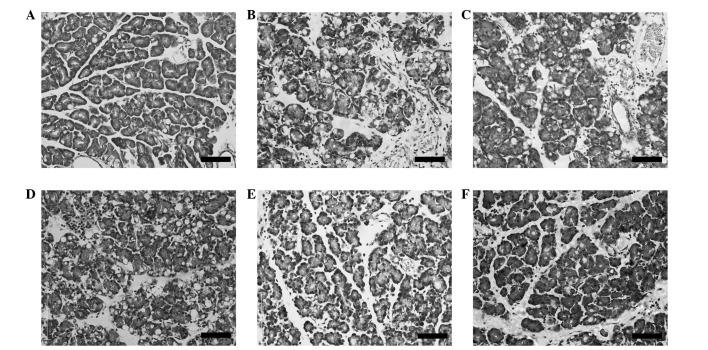
Hematoxylin and eosin (HE) staining of pancreatic tissue. (A) HE staining was detected in a control rat. (B-F) The tissues of CAP rats were obtained at 1, 3, 6, 12 and 24 h after cerulein treatment, respectively. Bar indicates 50 μm. CAP, cerulein-induced acute pancreatitis.

Rat pancreatic tissues were stained with HE for morphological examination. None of the control tissues showed characteristics of AP at any time point (Fig. 2A). CAP tissues showed evidence of interstitial edema, cellular vacuolation and inflammatory cell infiltration. The most severe interstitial edema and cellular vacuolation were observed at 6 h (Fig. 2D), whereas inflammatory cell infiltration in the interstitial space was most predominant at 12 h (Fig. 2E). Some recovery was apparent by 24 h after cerulein treatment, as pancreatic tissue appeared more similar to the control (Fig. 2F).

### Apoptosis assay during CAP

**Figure 3:**
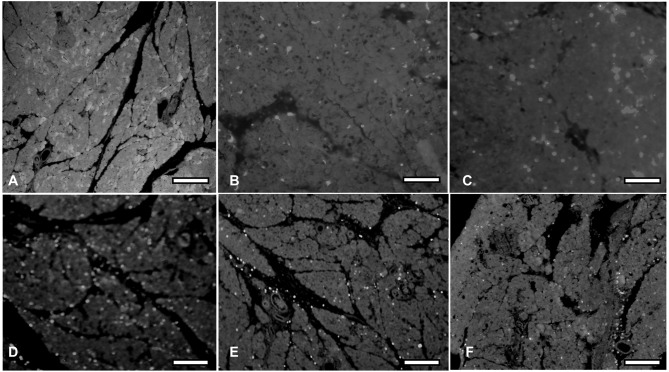
TUNEL staining of pancreatic tissue. (A) TUNEL staining was detected in control rats. (B-F) The tissues of CAP rats were obtained at 1, 3, 6, 12 and 24 h after cerulein treatment, respectively. Bar indicates 50 μm. CAP, cerulein-induced acute pancreatitis. TUNEL, terminal-deoxynucleotidyl-transferase-mediated dUTP nick-end labeling.

**Figure 4:**
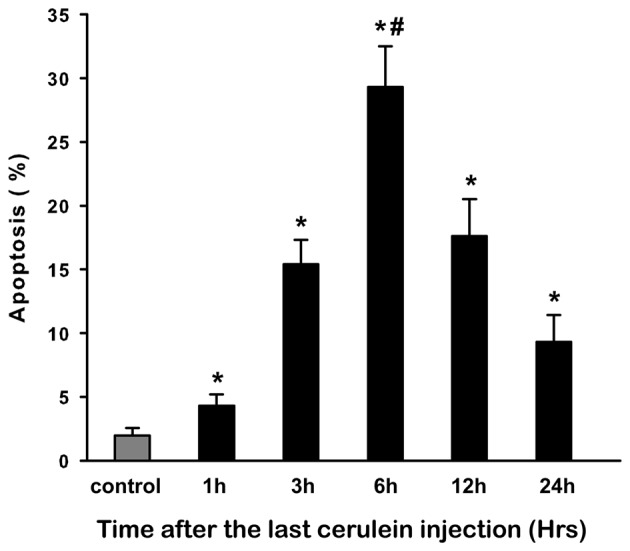
Apoptosis index of rat pancreatic tissue. The apoptosis index was based on TUNEL assay. Values are the means ± SE (n=6), ^*^P<0.05, vs. control. TUNEL, terminal-deoxynucleotidyl-transferase-mediated dUTP nick-end labeling.

Apoptosis in pancreatic acinar cells was determined by TUNEL assay (Fig. 3). The results of TUNEL assay showed that cerulein induced a time-dependent increase in apoptosis in comparison with control rats. We observed that AI was markedly increased at 3 h and peaked at 6 h (P<0.05). Although this increase began to decrease after 6 h, it was significantly increased at 24 h in comparison with the control (P<0.05; Fig. 4).

### Expression of caspase genes during CAP

**Figure 5:**
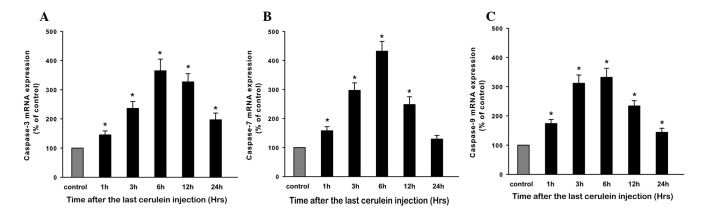
Comparison of relative mRNA expression for apoptotic genes by real-time RT-PCR. Data are expressed as the percentage of control. (A) Caspase-3 mRNA levels; (B) caspase-7 mRNA levels; (C) caspase-9 mRNA levels. Values are the means ±SE (n=6). ^*^P<0.05 vs. control.

In order to further understand the apoptotic signaling pathway, we determined the initiator caspase-9 and the effector caspase-3 and -7 mRNA expression by real time RT-PCR (Fig. 5). Compared with control rats, the mRNA levels of caspase-3, -7 and -9 in CAP rats showed a basically consistent change, in that all levels were markedly increased at 1 h and peaked at 6 h (P<0.05, respectively), then began to decrease and gradually returned to baseline at 24 h.

### Expression of GRP78 during CAP

**Figure 6:**
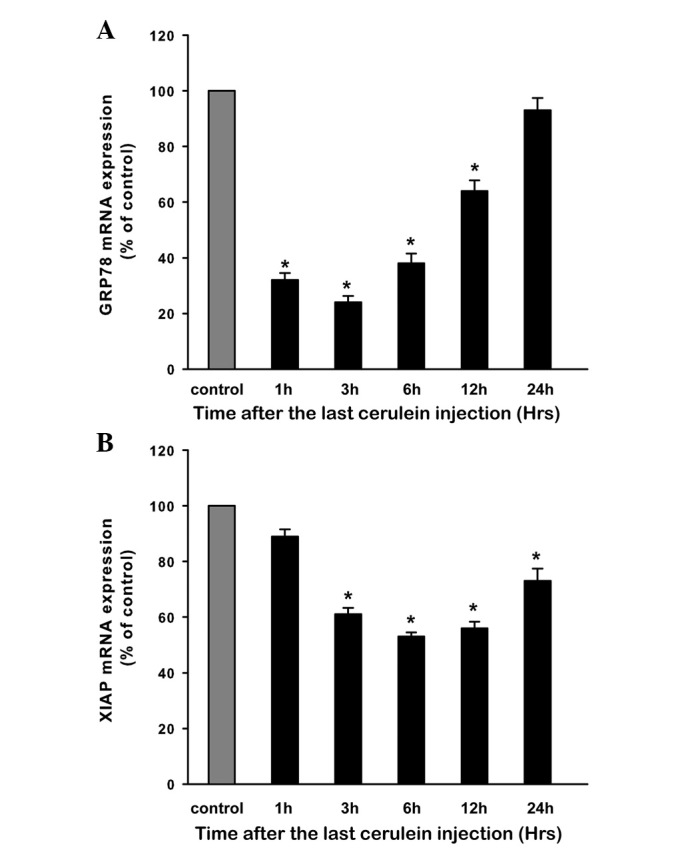
Comparison of relative mRNA expression for GRP78 and XIAP by real time RT-PCR. Data are expressed as a percentage of the control. (A) GRP78 mRNA levels; (B) XIAP mRNA levels. Values are the means ± SE (n=6). ^*^P<0.05 vs. control. XIAP, X-linked inhibitor of apoptosis protein; GRP78, 78-kDa glucose-regulated protein.

**Figure 7:**
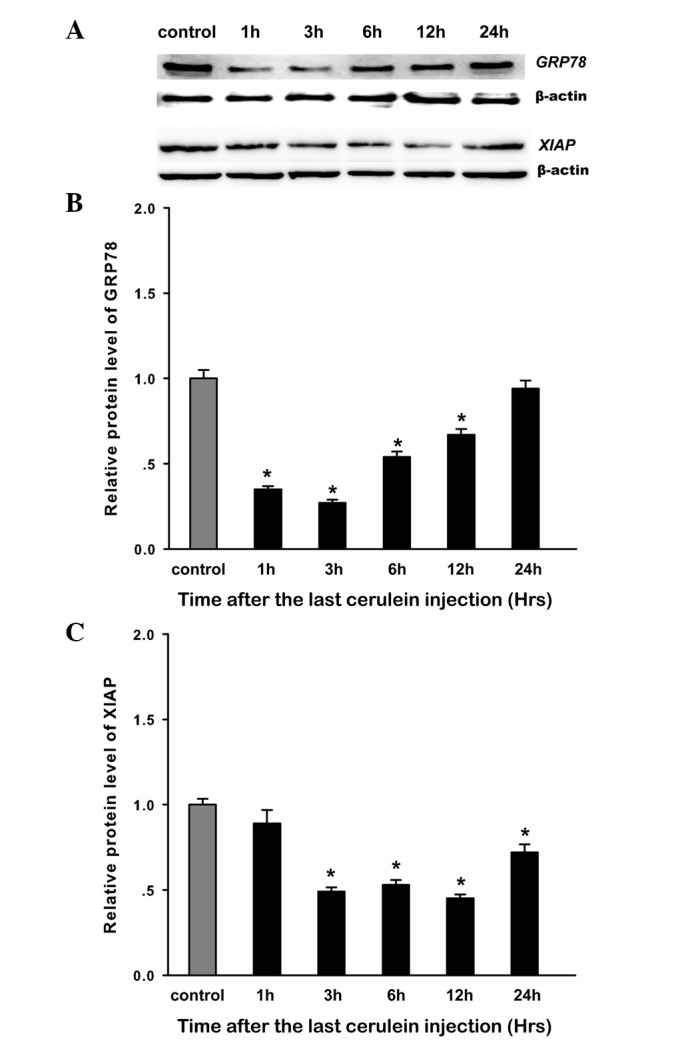
Comparison of relative protein expression for GRP78 and XIAP by western blot analysis. (A) Western blot results, β-actin was used as loading control. Relative protein expression of (B) GRP78 and (C) XIAP were determined by the semi-quantitative analysis. Values are the means ± SE (n=6). ^*^P<0.05 vs. control. GRP78, 78-kDa glucose-regulated protein; XIAP, X-linked inhibitor of apoptosis protein.

Expression of GRP78 mRNA in the pancreatic tissue was examined by real-time RT-PCR. Compared with control rats, the GRP78 mRNA level in CAP rats was decreased rapidly at 1 h and reached a low peak at 3 h (P<0.05). The level then began to increase, and returned to baseline at 24 h (Fig. 6A). The expression of GRP78 protein was evaluated by western blot analysis. The results showed a similar trend to mRNA change in the expression of GRP78 protein (Fig. 7A and B). A negative and significant correlation was demonstrated between GRP78 expression and the AI, and the expression of caspase-3, -7, and -9 (Pearson’s correlation of −0.757, −0.669, −0.871, −0.704, respectively, P<0.05 for all).

### Expression of XIAP during CAP

Expression of XIAP mRNA in the pancreatic tissue was examined by quantitative RT-PCR. In CAP rats, XIAP mRNA was decreased at 3 h and the low level was maintained up to 24 h in comparison with control rats (P<0.05; Fig. 6B). Western blot analysis showed a similar trend for mRNA change in the expression of XIAP protein (Fig. 7). XIAP expression was negatively correlated with the AI and the expression of caspase-3, -7 and -9 (Pearson’s correlation of −0.911, −0.950, −0.827, −0.855 respectively, P<0.05 for all).

## Discussion

AP is characterized not only by inflammation but also by parenchymal cell death (2,3,21). Apoptosis and necrosis are observed in clinical as well as experimental pancreatitis (5). Of note, the severity of experimental pancreatitis directly correlates with the extent of necrosis and inversely with that of apoptosis (4,22). Thus, apoptosis appears to be a beneficial response in the setting of AP. However, the mechanisms regulating apoptosis in AP are unclear. To explore the mechanisms regulating apoptosis response in AP, in the present study, we analyzed the apoptosis response in CAP, the most commonly used and well-characterized rat model of AP. Consistent with previous studies, the induction of CAP was demonstrated by the elevation of serum amylase activity, pancreatic edema, vacuolization, inflammation and apoptosis. However, little necrosis was present in CAP rats.

To explore the apoptotic signaling pathway during CAP, we analyzed the pro-apoptotic caspase genes by real-time PCR. We found the mRNA levels of the initiator caspase-9 and the effector caspase-3 and -7 were rapidly and significantly increased within 1 h and peaked at 6 h. Correspondingly, these increased caspase levels were consistent with AI in CAP rats, as calculated by TUNEL assay. These results indicated that the pro-apoptotic signaling pathway was activated in CAP.

To further study the potential role of GRP78 in CAP, we analyzed the expression of GRP78 by real time RT-PCR and western blotting. The results showed that the mRNA and protein levels of GRP78 were markedly downregulated in early CAP (within 1 h), although the expression levels returned to baseline at 24 h. It is notable that GRP78 was upregulated in the early stage of arginine-induced severe rat AP, which exhibited low apoptosis in pancreatic acinar cells (23). By contrast, downregulation of GRP78 expression was found in a cerulein-induced *in vitro* mild model of AP, which showed high levels of apoptosis in AR42J cells (24). Taken together, these findings suggest that GRP78 participates in the pathogenesis of AP, and it may regulate apoptosis during AP. Moreover, previous studies have indicated that GRP78 serves an anti-apoptotic role by forming complexes with caspase-7 and caspase-12 and preventing their activation and release (14,20). In this study, the downregulated expression of GRP78 was negatively correlated with the expression of caspase-3, -7 and -9 during CAP.

There are other data indicating that GRP78 plays an anti-apoptotic role, perhaps through interacting with XIAP (18), which is the most potent member of IAPs that negatively regulate caspase activation (13). Indeed, it has been indicated that XIAP was degraded rapidly and fully in rat CAP, and that its degradation caused caspase activation (22). In the present study, the mRNA and protein levels of XIAP were markedly downregulated within 3 h, which was negatively correlated with the caspase gene expression during CAP. Thus, these findings suggest that XIAP is an important caspase regulator in AP. In particular, high XIAP levels block caspase activation, whereas XIAP degradation facilitates caspase activation.

In conclusion, our study revealed that GRP78 and XIAP are downregulated in the process of CAP in rats. The expression changes of GRP78 and XIAP were negatively correlated with caspase gene expression and AI in CAP. These findings indicate that downregulation of GRP78 and XIAP was correlated with apoptosis through negatively regulating caspase activation during CAP in rats.

## Abbreviations

CAP: cerulein-induced acute pancreatitis
GRP78: 78-kDa glucose-regulated protein
XIAP: X-linked inhibitor of apoptosis protein
